# Supplementary figures and images for: Identification of miRNAs and Target Genes at Key Stages of Sexual Differentiation in Androdioecious Osmanthus fragrans
Source: Int J Mol Sci. 2022 Sep 8;23(18):10386. doi: 10.3390/ijms231810386 (PMC9499476; doi:10.3390/ijms231810386)

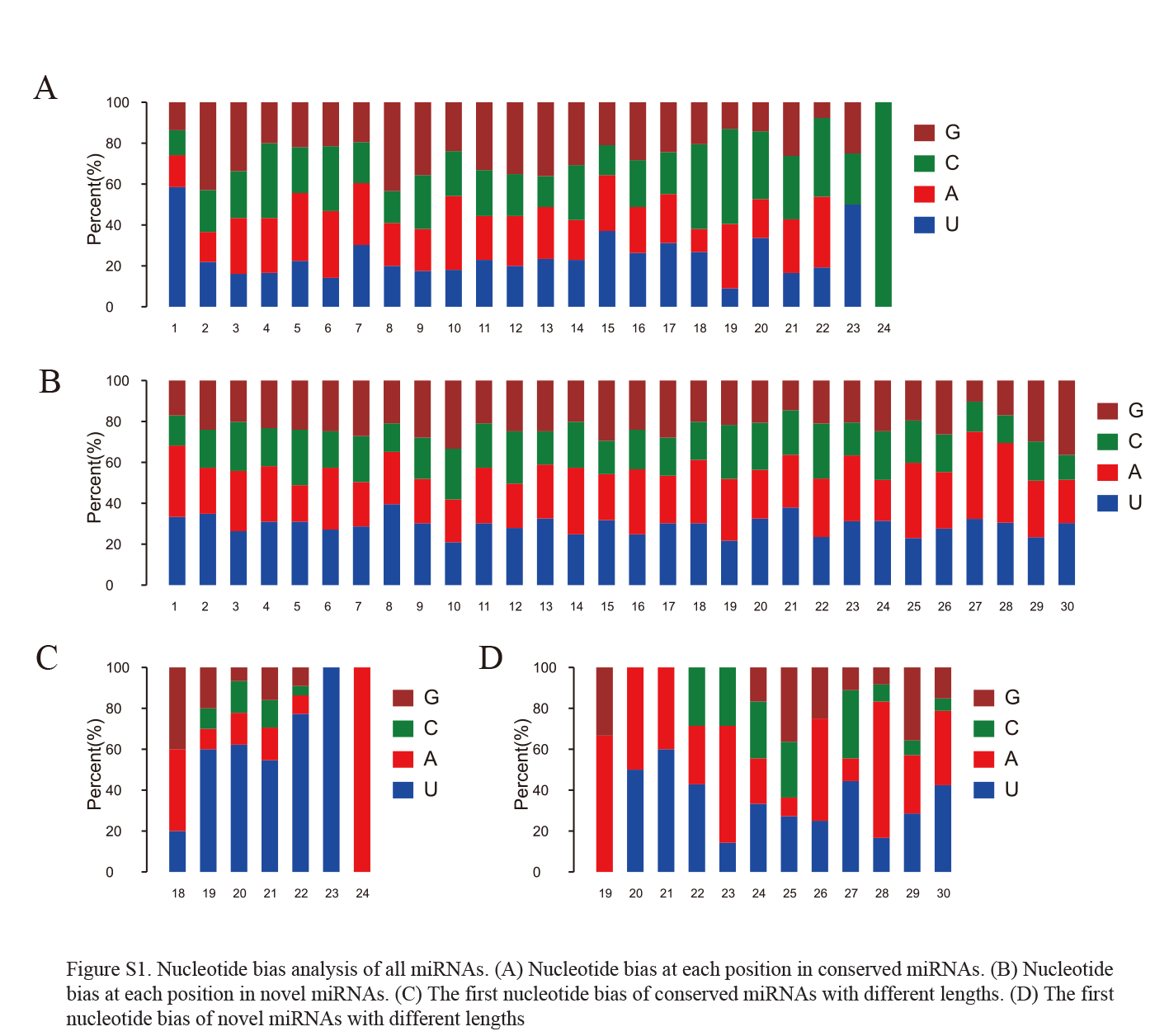

Supplement: Supplementary file 1 [file ijms-23-10386-s001.zip › Supplementary Files/Fig S1.tif]
